# Placental Protein 13: Vasomodulatory Effects on *Human* Uterine Arteries and Potential Implications for Preeclampsia

**DOI:** 10.3390/ijms25147522

**Published:** 2024-07-09

**Authors:** Mariacarmela Gatto, Milena Esposito, Michele Morelli, Silvia De Rose, Sveinbjorn Gizurarson, Hamutal Meiri, Maurizio Mandalà

**Affiliations:** 1 Department of Biology, Ecology and Earth Sciences, University of Calabria, 87036 Rende, Italy; mariacarmelagatto91@hotmail.it (M.G.); milenaesposito17@gmail.com (M.E.); 2 Department of Gynecology and Obstetrics, Hospital SS Annunziata, 87100 Cosenza, Italy; morellimichele122@gmail.com (M.M.); slvderose@gmail.com (S.D.R.); 3 Faculty of Pharmaceutical Sciences, University of Iceland, 101 Reykjavik, Iceland; sveinbj@hi.is; 4 Hylabs Ltd., Rehovot 7670606, Israel; hamutal62@hotmail.com; 5 TeleMarpe Ltd., Tel Aviv 6908742, Israel; 6 Department of Obstetrics, Gynecology and Reproductive Sciences, Larner College of Medicine, University of Vermont, Burlington, VT 05401, USA

**Keywords:** galectin, nitric oxide, cGMP, vascular tone, gestational hypertension

## Abstract

Placental protein 13 (PP13) exhibits a plasma concentration that increases gradually during normal gestation, a process that is disrupted in preeclampsia, which is characterized by elevated vascular resistance, reduced utero-placental blood flow, and intrauterine growth restriction. This study investigated PP13’s role in vascular tone regulation and its molecular mechanisms. Uterine and subcutaneous arteries, isolated from both pregnant and non-pregnant women, were precontracted with the thromboxane analogue U46619 and exposed to PP13 using pressurized myography. The molecular mechanisms were further investigated, using specific inhibitors for nitric oxide synthase (L-NAME+LNNA at 10^−4^ M) and guanylate cyclase (ODQ at 10^−5^ M). The results showed that PP13 induced vasodilation in uterine arteries, but not in subcutaneous arteries. Additionally, PP13 counteracted U46619-induced vasoconstriction, which is particularly pronounced in pregnancy. Further investigation revealed that PP13’s mechanism of action is dependent on the activation of the nitric oxide–cGMP pathway. This study provides novel insights into the vasomodulatory effects of PP13 on human uterine arteries, underscoring its potential role in regulating utero-placental blood flow. These findings suggest that PP13 may be a promising candidate for improving utero-placental blood flow in conditions such as preeclampsia. Further research and clinical studies are warranted to validate PP13’s efficacy and safety as a therapeutic agent for managing preeclampsia.

## 1. Introduction

Pregnancy is characterized by a cascade of physiological changes and adaptations aimed at supporting the growth and development of the fetus [1]. From conception to birth, the pregnant woman navigates a complex interplay of hormonal, metabolic, and immunological shifts, all orchestrated to nurture and sustain the developing life within. During this time, the maternal body provides the necessary nutrients, oxygen, and protection for the growing fetus to thrive. However, the marvel of pregnancy comes with inherent risks and challenges, highlighting the critical nature of this period [2]. Complications arising during pregnancy can have far-reaching implications, both impacting immediate health outcomes and predisposing individuals to a spectrum of chronic diseases later in life [3]. Therefore, ensuring maternal health during pregnancy is essential for the successful progression of gestation and for laying the foundation for the long-term well-being of both the mother and child.

The placenta is critical to a healthy pregnancy outcome since it takes on the multifaceted role of supporting fetal growth and ensuring the well-being of both the developing fetus and the mother [4]. Moreover, it also acts as an endocrine organ by synthetizing and secreting various proteins, including PP13, a member of the galectin family [5]. Secreted by placental syncytiotrophoblasts, PP13 enters maternal circulation as early as the 5th week of gestation [6]. In healthy pregnancies, PP13 plasma concentrations rise progressively throughout gestation, peaking at around 400 pg/mL by term [7]. The PP13 content of the placenta is approximately 2.5 mg by the end of gestation, thereby constituting about 7% of placental proteins [6].

The significance of PP13 lies in its association with preeclampsia (PE) [8], a hypertensive disorder of pregnancy, characterized by onset in the first trimester and often leading to maternal and perinatal morbidity and mortality [9]. Research indicates a stark decrease in PP13 levels in the maternal circulation during the first trimester of pregnancies complicated by P. This is in contrast to healthy pregnancies, where concentrations steadily increase [10]. Altered levels of PP13 mRNA and protein, as well as polymorphic variants found in women with PE, suggest a potential role for PP13 in the pathogenesis of this disorder [11,12]. PE poses significant risks to maternal and fetal health, including increased cardiovascular disease risk and improper vascular remodeling, which can lead to intrauterine growth restriction. Correct vascular remodeling during pregnancy is essential for maintaining adequate utero-placental blood flow, with the uterine circulation undergoing expansive remodeling to accommodate the increased demand for oxygen and nutrients required for fetal growth [13].

PP13 modulates several key processes that are critical for ensuring a successful pregnancy, including maternal immune responses to prevent maternal–fetal rejection [14], modulate oxidative stress and inflammation to mitigate oxidative damage and inflammatory responses [15,16], promote cytotrophoblast invasion into the myometrium, and ensure spiral artery remodeling [17,18]. Animal studies have also shown that PP13 modulates endothelial cell function [19,20], promotes angiogenesis, and facilitates the vascular remodeling necessary for proper placental and fetal development [21].

Despite these findings, our understanding of how PP13 affects the maternal vasculature during pregnancy is incomplete. Therefore, the objective of this study was to investigate the effects of PP13 on the vasculature of pregnant and non-pregnant women and to elucidate its mechanisms of action and potential implications in pregnancy-related vascular physiology. A comprehensive understanding of how PP13 affects maternal vessels is essential for elucidating the pathophysiology of pregnancy-related vascular disorders. This research may provide valuable insights into the diagnostic and therapeutic applications of PP13 in pregnancy-related disorders.

## 2. Results

**Figure 1:**
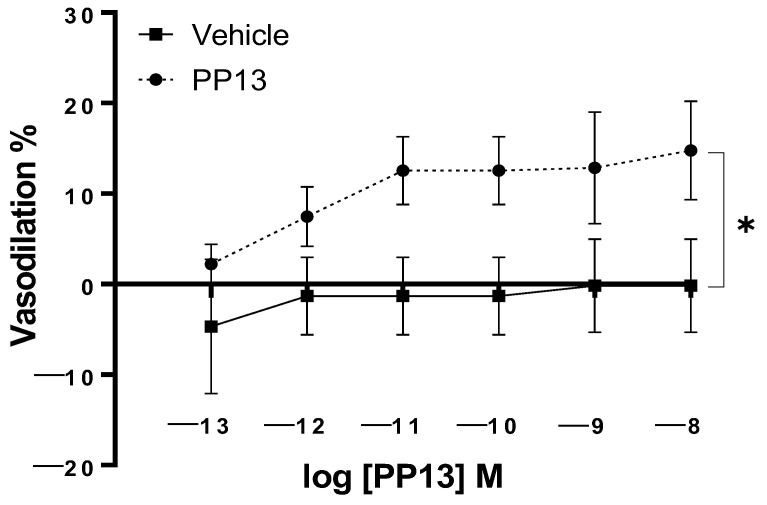
Effect of PP13 (vs. vehicle alone) on uterine arteries isolated from pregnant women. PP13 (dashed line, *n* = 6) induced vasodilation that could not be attributed to the vehicle (continuous line, *n* = 6), * *p* < 0.05.

PP13 and its vehicle were assessed for their effects on uterine arteries that were isolated from pregnant women. The results demonstrated a concentration-dependent vasodilatiory effect, with a maximal dilation of 14 ± 5.4% at a concentration of 10^−8^ M. The EC_50_ value for PP13 was calculated to be 1.7 × 10^−12^ M. In contrast, the administration of the vehicle did not induce any significant effect on arterial diameter (Figure 1). These findings indicate the potent vasodilatory action of PP13 and highlight the biological activity of PP13 in regulating uterine vascular tone.

**Figure 2:**
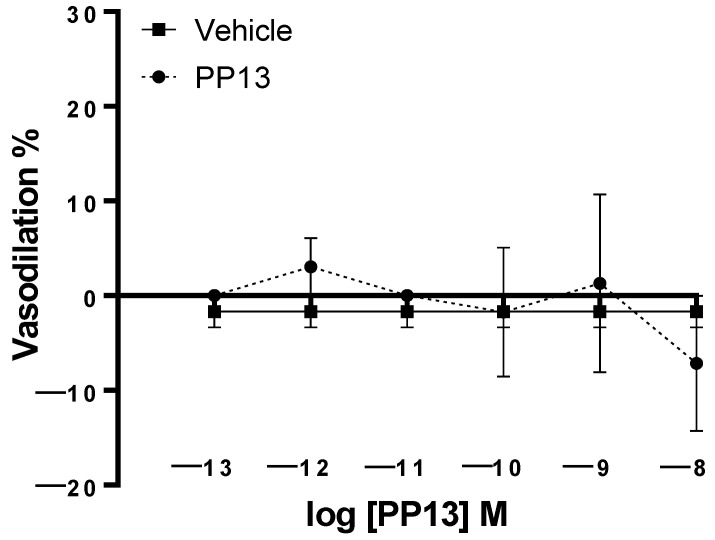
Effect of PP13 and its vehicle on subcutaneous arteries isolated from pregnant women. Neither PP13 (dashed line, *n* = 6) nor its vehicle (continuous line, *n* = 6) had any effect.

PP13 and its vehicle were also evaluated on subcutaneous arteries obtained from pregnant women. Unlike uterine arteries, neither PP13 nor its vehicle significantly affected subcutaneous arterial function (Figure 2). These results suggest the specificity of PP13 action on uterine arteries in this experimental context.

**Figure 3:**
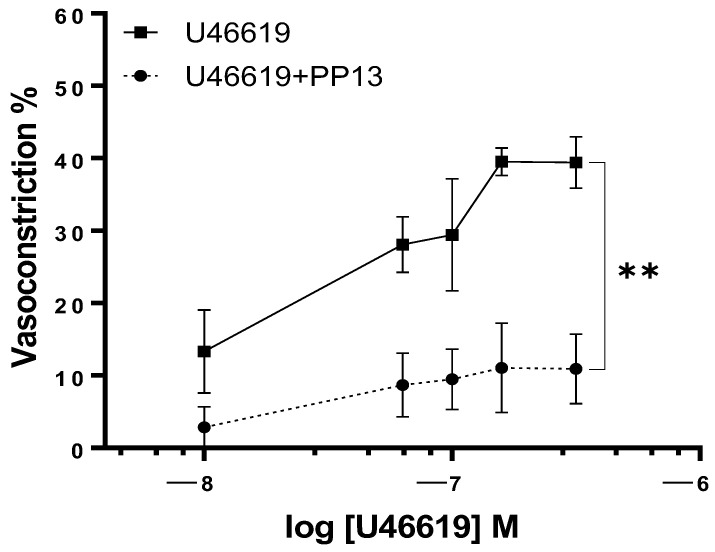
Effect of PP13 on vasoconstriction in isolated uterine arteries taken from pregnant women. Vessels were constricted with U46619 (*n* = 6) in the absence vs. presence of PP13 at 10^−8^ M (U46619 + PP13, *n* = 6), ** *p* < 0.01.

Given the evidence for a vasodilatory role for PP13 in uterine arteries, we sought to investigate its potential counteractive effects against the potent vasoconstrictor U46619, a thromboxane receptor agonist. Uterine arteries, isolated from pregnant women, were exposed to U46619 in the presence and absence of PP13. As anticipated, U46619 induced a concentration-dependent contraction of uterine arteries. However, intriguingly, the contraction was significantly attenuated in the presence of PP13 (Figure 3). The maximum U46619-contraction was reduced by 71% (from 40 ± 6.3% to 12 ± 4.8%) by PP13 addition. These findings highlight the vasomodulatory potential of PP13 in regulating uterine vascular tone.

**Figure 4:**
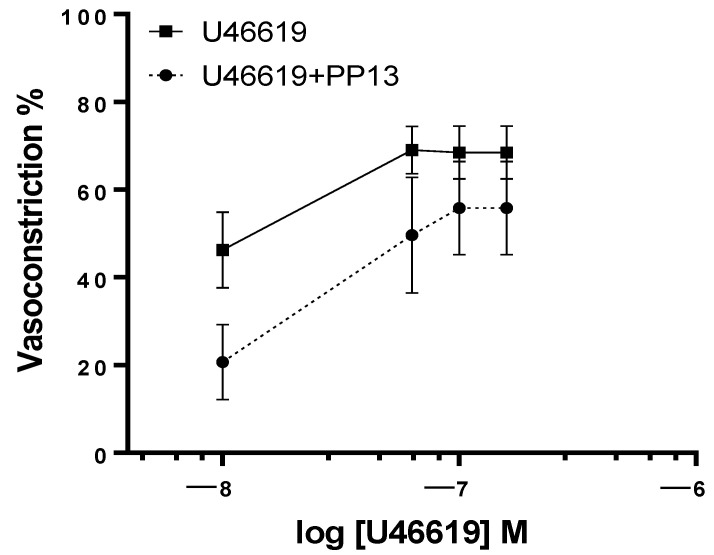
Effect of PP13 on vasoconstriction in uterine arteries isolated from non-pregnant women. Vessels were constricted with the vasoconstrictor U46619 (*n* = 6) in the absence vs. presence of PP13 at 10^−8^ M (U46619 + PP13, *n* = 6).

To determine whether PP13’s effect on vascular tone is dependent on pregnancy, we conducted experiments using uterine arteries isolated from non-pregnant women. Interestingly, PP13 reduced U46619-induced contraction in these arteries as well, but the change was not significant, as depicted in Figure 4. PP13 was less effective in attenuating U46619 contraction in non-pregnant women than in pregnant women. In uterine arteries taken from non-pregnant women, PP13 reduced the maximum U46619-contraction by only 32% (from 72 ± 9.2% to 49 ± 14.6%). Thus, its vasodilatory effect is potentiated in the pregnant state.

**Figure 5:**
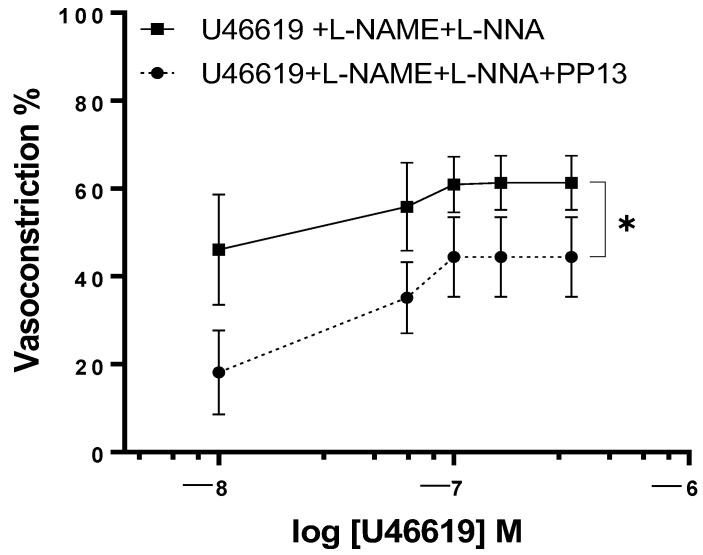
The effects of inhibition of nitric oxide production on PP13 action. Uterine arteries isolated from pregnant women with and without preincubation in NOS inhibitors (L-NAME + L-NNA) were constricted with U46619 in the absence (U46619 + L-NAME + L-NNA, *n* = 6) vs. presence of PP13 at 10^−8^ M (U46619 + L-NAME + L-NNA + PP13, *n* = 6), * *p* < 0.05.

To elucidate the mechanism by which PP13 counteracts U46619-induced contraction in uterine arteries isolated from pregnant women, we investigated whether PP13 acts through the nitric oxide pathway. To this end, uterine arteries were preincubated with specific inhibitors of the enzyme NOS, L-NAME, and L-NNA and then exposed to U46619 in either the presence or absence of PP13 (Figure 5). Although the concentration–response curve for U46619 contraction remained significantly (*p* < 0.05) reduced, the counteracting effects of PP13 were much lower than those reported in Figure 3 (*p* < 0.01). 

This indicates that the inhibition of NO production in part prevents the observed antagonistic action of PP13 on U46619-induced contraction and suggests that PP13 may exert its vasomodulatory effects by modulating the production or availability of nitric oxide, most likely via the endothelium.

**Figure 6:**
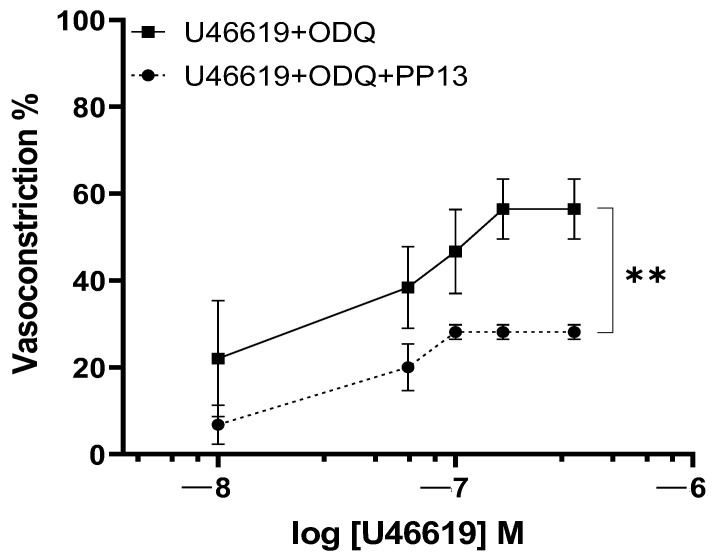
The inhibitory effects of guanylate cyclase on PP13 action. Uterine arteries taken from pregnant women were deprived of cGMP production by ODQ and treated with the vasoconstrictor U46619 in the presence (U46619 + ODQ + PP13, *n* = 6) or absence (U46619 + ODQ, *n* = 6) of PP13 (10^−8^ M), ** *p* < 0.01.

To further elucidate the transduction pathway through which PP13 exerts its effects, we investigated its action in the presence of a specific inhibitor of guanylate cyclase, ODQ. Uterine arteries were preincubated with ODQ to inhibit the production of the nucleotide cGMP. In the presence of ODQ, the dose–response curve for U46619 contraction in PP13 treated vessels remained significantly reduced (*p* < 0.01), as shown in Figure 6. These results suggest that PP13’s mechanism of action is cGMP-independent and most likely affects vascular smooth muscle.

## 3. Discussion

This study investigated the impact of PP13 on human vasculature, focusing specifically on both uterine (reproductive) and subcutaneous (systemic) arteries isolated from pregnant and non-pregnant women. The results revealed several key findings: (1) PP13 exhibited pregnancy-specific vasodilatory action in uterine arteries, while no such effect was observed in subcutaneous arteries, indicating the selectivity of PP13 action on the reproductive vasculature; (2) PP13 mitigated the vasoconstriction induced by U46619, which was particularly pronounced during pregnancy; and (3) this was achieved partially via its stimulation of the NO pathway.

This study was performed on small subcutaneous and uterine arteries with a lumen diameter of less than 250 μm. These vessels are recognized as pivotal regulators of vascular resistance, thereby controlling blood flow to downstream organs and tissues. Specifically, uterine arteries are responsible for supplying blood to the uterus, a role which becomes even more critical during pregnancy as they orchestrate circulation to the feto-placental unit. 

It is well established that profound adaptations must occur in the structure and function of the maternal uterine circulation during pregnancy to sustain the metabolic demands of fetal development and optimize utero-placental blood flow [13]. Regulated by the complex interplay of hormonal, mechanical, and paracrine factors, these include angiogenesis, growth, vasodilation, and alterations in vascular compliance and tone [22]. Together, these changes lead to the substantial and progressive enhancement of utero-placental blood flow, which increases approximately 10–20 times above non-pregnant uterine flow levels. Studies utilizing Doppler ultrasound and MRI have reported uterine blood flow values of around 50 mL/min in non-pregnant women, which escalate to levels of 500–600 mL/min during late pregnancy [23,24,25]. This significant increase underscores the dynamic nature of the tissue and the vascular adaptations necessary to sustain fetal growth and development.

The placenta assumes a central role in orchestrating physiological changes within the maternal uterine vasculature during pregnancy through its secretion of hormones, growth factors, and cytokines that exert endocrine and paracrine effects [26,27]. Given the marked decrease it induces in the plasma concentration in PE [8], evaluating the effects of PP13 on the maternal vasculature is paramount. Notably, preeclampsia is linked to impaired uterine vascular remodeling, resulting in elevated vascular resistance and reduced utero-placental blood flow [14]. 

The effects of PP13 on subcutaneous and uterine arteries were assessed using pressure myography, a well-established ex vivo technique renowned for its ability to closely replicate in vivo physiological conditions. This method, which allows for the examination of vascular responses in the presence of a true transmural pressure, is more reflective of the in vivo situation than traditional wire myography. 

Our findings underscored the specificity of PP13 action on uterine arteries. This aligns with PP13’s role as a placental protein that is primarily involved in reproductive processes. Although prior studies demonstrated PP13-induced vasodilation in rat uterine arteries, as well as in mesenteric arteries [19,20,27], this study is the first to examine PP13’s effects on human uterine arteries, a critical distinction given that preeclampsia occurs exclusively in humans.

Moreover, PP13’s ability to counteract U46619-induced vasoconstriction, which is particularly enhanced during pregnancy, may be facilitated by gestation-associated vascular conditions, such as increased endothelial NO levels in uterine arteries [28]. The endothelial cells lining the lumen of all blood vessels, including uterine arteries, are surrounded by vascular smooth muscle cells (VSMCs). These endothelial cells serve as sensors and effectors of multiple inputs, such as pressure, flow (shear stress), and vasoactive substances present in the bloodstream. In turn, they release various mediators, including NO, prostaglandins, and other hyperpolarizing factors, which induce the relaxation of VSMCs (endothelium-dependent dilation). 

Consistent with previous findings in rats, our results indicate that the vasodilatory actions of PP13 in human vessels are mediated through NO, indicating likely endothelium-dependent action and that this effectively opposes the action of vascoconstrictor influences [29,30]. 

During pregnancy, the expression of endothelial nitric oxide synthase (eNOS) undergoes significant upregulation, primarily from elevated estrogens [31] and shear stress [32]. This upregulation is pivotal for augmenting endothelial vasodilatory influence on the adjacent VSM. As a result, uterine vascular resistance is reduced, leading to enhanced blood flow to the uterus and the feto-placental unit. 

The effects of NO are conventionally attributed to the activation of soluble guanylyl cyclase, which leads to the formation of cyclic guanosine-3,5-monophosphate (cGMP). However, our findings indicate that PP13-NO-induced vasodilation operates through a pathway that is independent of cGMP. This suggests the existence of alternative mechanisms that contribute to the vasodilatory effects of nitric oxide beyond the traditional NO-cGMP pathway. Emerging evidence has shed light on these alternative signaling pathways. For instance, nitric oxide has been shown to directly stimulate potassium channels [33] and calcium channels [34]. These findings underscore the complexity of nitric oxide signaling and its diverse physiological implications, paving the way for further exploration into the intricate and interactive mechanisms underlying vascular regulation. 

Although this study provided some insight into the role of NO in PP13’s vasodilatory effect, it did not delve into the precise molecular mechanisms underlying this action and further investigations are warranted to elucidate the specific pathways involved. Another limitation to consider is that our studies focused on isolated arteries, which cannot fully replicate the complex vascular environment in vivo. 

In conclusion, the insights gained from this study offer valuable groundwork for future investigations into a potential therapeutic role for PP13 in treating preeclampsia, a condition that affects approximately 8% of women worldwide and is still without a definitive cure. Clinical studies are warranted to ascertain the efficacy and safety of PP13 administration in preeclamptic women, ensuring positive outcomes for both maternal and fetal health.

## 4. Material and Methods

### 4.1. Patient Recruitment and Selection

This study involved recruiting patients undergoing cesarean delivery or hysterectomy, all within reproductive age (ranging from 20 to 45 years). A total of 17 patients participated, comprising 5 non-pregnant individuals and 12 pregnant patients with normal pregnancies. Patients were informed about the project upon hospitalization and provided with detailed explanations. Those who agreed to participate underwent a health assessment and provided informed consent. Ethical approval was obtained from the local Research Ethics Committee [approval number: Prot. 8177, 11 February 2022)] in adherence with the principles outlined in the Declaration of Helsinki. Exclusion criteria included twin pregnancies and pre-existing conditions, such as chronic arterial hypertension, autoimmune, renal, or hepatic diseases, ensuring that enrolled patients were free from debilitating pathologies.

### 4.2. Samples Collection

Samples of subcutaneous fat and myometrium (~1 cm^3^) were obtained from consenting healthy women at the Department of Gynecology and Obstetrics, Annunziata Hospital of Cosenza, Italy. Tissues were immediately immersed in cold HEPES–physiological salt solution (HEPES-PSS), kept on ice, and transported within 30 min to the vascular physiology laboratory for arteriole isolation.

### 4.3. Vessel Preparation

In the laboratory setting, tissues were initially rinsed with fresh cold HEPES-PSS to ensure the removal of any blood residue from the sample surface. Subsequently, the tissues were delicately transferred into small glass dissecting dishes (90 mm) filled with cold HEPES-PSS solution. Once in the dish, they were carefully positioned at the bottom and secured in place using insect pins. The bottom of the dishes was covered with a smooth layer of silicone elastomer (Sylgard 184) to provide a stable working surface. 

Arterioles were dissected under a stereomicroscope (Leica MZ 12.5 Ergo) utilizing fine forceps (Dumont #55) and sharp Vannas spring scissors (Fine Science Tools, Foster City, CA, USA), ensuring the preservation of their structural integrity and minimizing any potential damage.

### 4.4. Pressure Myography

Artery segments, cut free of surrounding adipose and connective tissue, were isolated and transferred to a pressure myograph chamber (Instrumentation and Model Facility, University of Vermont, Burlington, VT, USA). Cannulation was conducted at both ends of the vessel between two opposing glass cannulas filled with HEPES-PSS, requiring the use of forceps with fine tips to ensure precision. Arterial segments of a sufficient length, approximately 3 mm, were mounted to ensure that there was an undamaged segment between the two cannulas. 

To elaborate on the cannulation process, one end of the vessel was mounted and tied onto glass pipettes using thin nylon suture thread, and any residual blood within the lumen was gently flushed out with the solution. Subsequently, the other end of the vessel was tied onto the downstream cannula, which was closed off to establish a “blind sack” preparation. Meanwhile, the inflow cannula, connected to a pressure-servo system (Living Systems Instrumentation, Burlington, VT, USA), was adjusted to a physiological pressure level of 50 mmHg to mimic in vivo conditions. This pressure-servo system was coupled with a pressure transducer to monitor the pressure applied to the vessels throughout the experiment. Each vessel was evaluated for leaks and, if present, discarded as these can influence vasodilation and vasoconstriction. The preparation was continuously superfused with HEPES-PSS, warmed to 37 °C and possessing a pH of 7. Arterial lumen diameter was continuously monitored using video microscopy, coupled with specialized data-acquisition software (Ionoptix 6.3.4.69, Westwood, MA, USA), to track the relative positions of the inner and/or outer vascular walls.

### 4.5. Experimental Protocol

Arteries were tested for viability using vasoactive agents U46619 (constrictor) and acetylcholine (dilator). Non-responsive vessels were excluded. Functioning arteries were equilibrated for 30–45 min and subjected to PP13 testing using two protocols: (1) vessels pre-constricted with U46619 were exposed to increasing PP13 concentrations (10^−13^–10^−8^ M); and (2) a concentration–response curve for U46619 was generated in the absence vs. presence of PP13 (10^−8^ M, pre-incubated for 1 h). At the end of the experiment, vessels were superfused with HEPES-PSS without calcium plus the phosphodiesterase inhibitor, papaverine (10^−4^ M), to induce maximal vasodilation and to measure the maximal vessel diameter. The underlying molecular mechanism of PP13 action was investigated using the following pharmacological inhibitors: (1) Nω-nitro-L-arginine methylester (L-NAME 10^−4^ M) and Nω-nitro-l-arginin (LNNA 10^−4^ M) for the eNOS enzyme, and (2) 1H-[1,2,4]oxadiazolo[4,3-a]quinoxalin-1-one (ODQ) for the guanylate cyclase enzyme. Uterine arteries were preincubated with inhibitors for 20 min and then constricted with U46619 before undergoing testing with PP13.

### 4.6. Chemicals and Solutions

The HEPES-PSS solution consisted of the following components in mM: 141.8 NaCl; 4.7 KCl; 1.7 MgSO_4_; 0.5 EDTA; 2.8 CaCl_2_; 10.0 HEPES; 1.2 KH_2_PO_4_; 5.0 glucose. The solutions were prepared in deionized water and titrated with sodium hydroxide to a physiologic pH of 7. Chemicals were purchased from Sigma-Aldrich (Milan, Italy), Fisher Scientific (Milan, Italy), and Cayman Chemical Co. (Hamburg, Germany), unless otherwise specified. All drugs tested were administered from stock solutions, prepared daily, while PP13, U46619 and ODQ stock solutions were stored in aliquots and were thawed as needed.

Recombinant PP13 was obtained from Protein Laboratories Hylabs, Ltd. (Rehovot, Israel). in collaboration with HyLaboratories Ltd. (Rehovot, Israel). The protein was dissolved in sterile water (vehicle).

### 4.7. Statistical Analyses

The vasodilatory effect of PP13 was expressed as a percentage of maximal diameter, which was determined in the presence of the relaxing solution. EC_50_ was calculated as the concentration that induced 50% of the maximum response. The area under the concentration–response curve (AUC) was compared across experimental conditions. Data were expressed as means ± standard error mean (SEM), with statistical significance set at *p* < 0.05 using an unpaired Student’s *t*-test.

## Data Availability

The data presented in this study are available on request from the corresponding author.
